# Carbopol/Chitosan Based pH Triggered *In Situ* Gelling System for Ocular Delivery of Timolol Maleate

**DOI:** 10.3797/scipharm.1001-06

**Published:** 2010-10-05

**Authors:** Swati Gupta, Suresh P. Vyas

**Affiliations:** 1 Nanomedicine Research Center, Department Of Pharmaceutics, I.S.F. College Of Pharmacy, Moga 142 001 (PB), India; 2 Drug Delivery Research Laboratory, Department of Pharmaceutical Sciences, Dr. Hari Singh Gour Vishwavidyalaya, Sagar (MP) 470 003, India

**Keywords:** Ocular delivery, *In situ* gelation, Timolol maleate, Glaucoma, Sustained delivery

## Abstract

The poor bioavailability and therapeutic response exhibited by conventional ophthalmic preparations due to rapid precorneal elimination, dilution and nasolacrimal drainage of the drug may be vanquished by the use of *in situ* gelling systems that are instilled as drops in to the eye and undergo a sol-gel transition in the cul-de-sac. Timolol eye drops may cause systemic side effects in glaucoma patients due to absorption of the drug into systemic circulation. *In situ* gelling system of this drug can provide localized effect with reduced contraindications, improved patient compliance and better therapeutic index. The present work describes the formulation and evaluation of an ophthalmic delivery system of an antiglaucoma drug, timolol maleate (TM) based on the concept of pH-triggered *in situ* gelation. Polyacrylic acid (carbopol) was used as the gelling agent in combination with chitosan (amine polysaccharide), which was acted as a viscosity-enhancing agent. Formulations were evaluated for pH, viscosity, gelling capacity and drug content. The 0.4% w/v carbopol/0.5% w/v chitosan based *in situ* gelling system was in liquid state at room temperature and at the pH formulated (pH 6.0) and underwent rapid transition into the viscous gel phase at the pH of the tear fluid (lacrimal fluid) (pH 7.4). The *in vitro* drug release and *in vivo* effects of the developed *in situ* gelling system were compared with that of Glucomol® (a 0.25% TM ophthalmic solution), 0.4% w/v carbopol solution as well as liposomal formulation. The results clearly demonstrated that developed carbopol-chitosan based formulation was therapeutically efficacious and showed a fickian (diffusion controlled) type of release behaviour over 24 h periods. The developed system is thus a viable alternative to conventional eye drops and can also prevent the rapid drainage as in case of liposomes.

## Introduction

Conventional liquid ophthalmic formulations demonstrate low bioavailability because of a constant lacrimal drainage in the eye. The normal drainage of an instilled drug dose commences immediately upon instillation and is essentially completed within 5 min [1]. Typically ophthalmic bioavailabilities of only 1–10% are achieved due to the short precorneal residence time of ophthalmic solutions [2]. Consequently, there is a need for frequent instillation of concentrated solutions to achieve the desired therapeutic effect [3]. Moreover, systemic absorption of the drug drained through the nasolacrimal duct may result in some undesirable side effects [4]. To overcome these problems various ophthalmic vehicles, such as viscous solutions, ointments, gels or polymeric inserts have been investigated in an attempt to extend the ocular residence time of medications for topical application to the eye [5]. The corneal contact time has been increased to varying degrees by these vehicles. But, they have not been unanimously accepted, because of blurred vision (e.g. ointments) or lack of patient compliance (e.g. inserts). As a result, good ocular bioavailability following topical delivery of a drug to the eye remains a challenge and yet to be resolved [6]. Timolol eye drops are commonly used to treat the elevated intraocular pressure (IOP) in glaucoma. Due to its systemic absorption timolol may produce systemic cardiac, respiratory, and central nervous system side effects especially in patients with predisposing factors [7, 8]. Systemic side effects of timolol contributed to the death of more than 30 patients in the United States during 1978 to 1985 [9]. Because of the risk of side effects, one fourth of patients receive other treatments.

From the point of view of patient acceptability, a liquid dosage form that can sustain drug release and remain in contact with the cornea of the eye for extended periods of time is ideal. If the precorneal residence time of a drug could be ameliorated from 5 min to one or two hours, then improved local bioavailability, reduced dose concentrations, less total drug, improved patient acceptability and reduced dosing frequency may be achieved. To furnish these properties, delivery systems based on the concept of *in situ* gel formation can be developed. Such delivery systems consist of phase transition systems that are instilled in a liquid form and shift to the gel or solid phase once in the cul-de-sac of the eye.

We suggest the use of *in situ* gel forming systems based on the combination of carbopol and chitosan. Carbopol is a polyacrylicacid (PAA) polymer, which shows a sol to gel transition in aqueous solution as the pH is raised above its pK_a_ of about 5.5 [10]. Chitosan, an amine-polysaccharide is also pH dependent, cationic polymer. Neutralization of chitosan aqueous solutions to a pH exceeding 6.2 systematically leads to the formation of a hydrated gel like precipitate [11]. When these two polymeric solutions are combined, the gel strength in the physiological condition could be significantly enhanced.

Thus, the aim of this study is to develop an *in situ* gelling formulation of TM based on the gelling properties of the carbopol-chitosan combinations with respect to their concentrations in the simulated tear fluid (STF, pH 7.4), and to evaluate the influence of the developed formulation on the experimentally induced IOP. Also, the liposomal formulations were prepared, optimized and compared with the developed *in situ* gelling system to check the comparative efficacy of the developed system. This new formulation could prove to be a novel ocular dosage form able to prolong the residence time and to control the release of drug when administered into the eye.

## Results and Discussion

### I. Preparation and optimization

Buffers play a pivotal role in formulating ophthalmic drops. They contribute significantly to chemical stability and clinical response and also influence the comfort and safety of the product. The studies in various buffer solutions indicated that the chitosan was soluble in acetate buffer of pH 4.6 at the desired concentration (0.5% w/v). The use of carbopol in *in situ* gelling systems is substantiated by the property of its aqueous solutions to transform into a stiff gel when the pH is raised [12]. However, the concentration of carbopol required to form stiff gel results in highly acidic solutions, which are not easily neutralized by the buffering action of the tear fluid [13, 14]. A reduction in carbopol (anionic polymer) concentration without compromising the gelling capacity and rheological properties of the delivery system was achieved by the addition of viscosity enhancing polymers such as chitosan. Autoclaving of polymeric solutions and terminal sterilization by UV irradiation had no effect on the pH, gelling capacity and viscosity of the formulations (data not shown). Several polymers, demonstrating phase transition due to changes in their micro-environment are being investigated. Among them are Poloxamer 407 [15] and tetronics [16], whose solution viscosity increases upon enhancing the temperature to that of the eye, cellulose acetophthalate (CAP) latex [17] coagulates when its native pH of 4.5 is raised by the tear fluid to pH 7.4 and Gelrite^®^, a polysaccharide which gels in the presence of mono or divalent cations [18]. However most of these vehicles are characterized by a high polymer concentration (25% poloxamer, 30% CAP), which is not well tolerated by the eye. In order to reduce the total polymer content and improve the gelling properties, Joshi et al. [19] first used the combination of polymers in the delivery system.

It was observed that the formulation ISGF_1_ (0.2% w/v carbopol and 0.5% w/v chitosan) was in liquid and clear solution state at room temperature (25°C) and at the pH formulated; however this could not form strong gel at the pH of STF (pH 7.4). On the other hand, formulation ISGF_4_ (0.5% w/v carbopol and 0.5% w/v chitosan) was in turbid solution state (Table 1). This might be due to formation of stiff gel even at room temperature and pH formulated. The formulations ISGF_2_ (0.3% w/v carbopol and 0.5% w/v chitosan) and ISGF_3_ (0.4% w/v carbopol and 0.5% w/v chitosan) retained liquid state (clear solutions) at room temperature and at the pH formulated and gelled upon exposure to pH of STF.

The loading of drug was found to be increased, when the carbopol concentration in the formulation was enhanced, which may be attributed to the charge based interaction between timolol and carbopol (Table 1). Although ISGF_4_ showed better drug payload and gelling capacity at physiological pH, it could not be considered as optimum formulation due to gel formation at room temperature and pH formulated. However, formulation ISGF_3_ was selected as optimum on the basis of drug content considering its satisfactory attributes of viscosity and gelling capacity at room temperature (Table 1). The pH of the optimized formulation ISGF_3_ was recorded to be 6. Liposomes were prepared using cast film method and compared with *in situ* gelling system (ISGF_3_). PC:CH molar ratio 7:3 (formulation LF_3_) was found to be optimum on the basis of percent drug entrapment (33.2±2.4) (Table 2). Whereas, other liposomal formulations (LF_1_, LF_2_, LF_4_, LF_5_) showed lower level of drug entrapment, when compared to LF_3_. Hence, LF_3_ was selected for further studies. The pH, clarity, gelling capacity and viscosity data of optimized formulation ISGF_3_ indicate that formulation might improve patient compliance during administration to the eye. In addition, the developed gel demonstrated adequate strength when it was pressed with a pair of fine forceps, indicating that it could withstand the low shear forces likely to be encountered in the cul-de-sac of the eye. Thus, carbopol/chitosan like vehicles could be less susceptible to drainage from the eye as seen in the case of ophthalmic solutions, and could have long residence time.

### II. Rheological studies

**Fig. 1.**
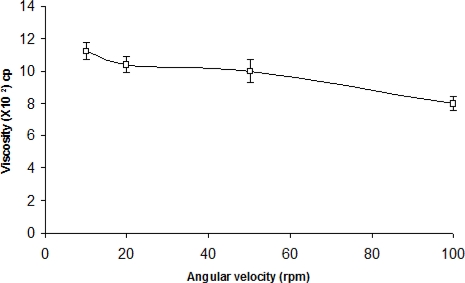
Rheological profile of pH triggered in situ gelling system (ISGF3) at pH 6.0 (n=6) (mean±sd)

**Fig. 2.**
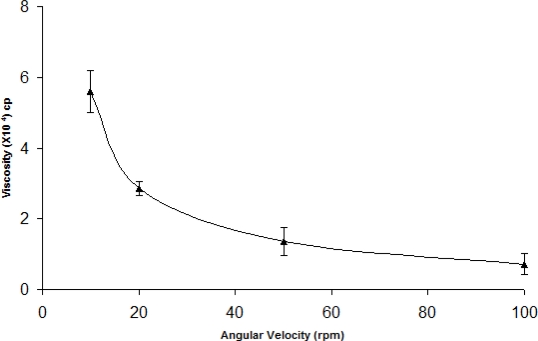
Rheological profile of pH triggered *in situ* gelling system (ISGF_3_) at pH 7.4 (n=6) (mean±sd)

The administration of ophthalmic preparations should influence as little as possible the pseudoplastic character of the precorneal tear film. Since the ocular shear rate is very large ranging from 0.03 s^−1^ during interblinking periods to 4250–28500 s^−1^ during blinking, viscoelastic fluids with a viscosity that is high under conditions of low shear rate and low under conditions of high shear are preferred [13]. The optimized formulation was subjected to shear thinning and an increase in shear stress resulted in an increase in angular velocity (pseudoplastic rheology). At pH 6.0, the formulation (ISGF_3_) was in a liquid state and exhibited low viscosity (Fig. 1). When the pH was increased to 7.4 (pH of the STF), liquid or solution state was transformed into the gel of high viscosity (Fig. 2).

### III. In vitro drug release studies

**Fig. 3.**
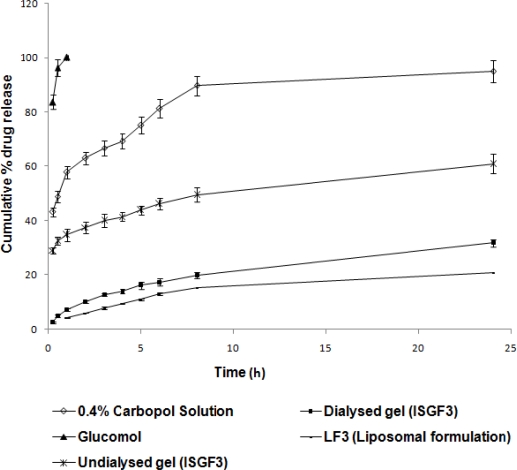
*In vitro* release of TM from different formulations (n=6) (mean±sd)

The cumulative percent of TM released as function of time from various formulations is shown in Fig. 3. In the case of TM containing 0.4% carbopol solution (without chitosan), the drug release was recorded to be about 43.2% after 15 minutes. Moreover, 89.7% of TM was released from the carbopol solution within 8 h. Whereas, TM containing 0.4% carbopol/0.5% chitosan solution (ISGF_3_) showed significantly lower drug release (p<0.05) i.e., only 2.6 and 29.1% of TM was released in the first 15 minutes, approximately 31.8 and 60.9% of TM was released after 24 h from dialysed and undialysed gel of formulation ISGF_3_ respectively and 20.8% of TM was released from LF_3_ after 24 h with the release profile continued thereafter (data not shown). The results indicated that the 0.4% carbopol/0.5% chitosan mixture was found to be more efficient in retaining drug and preventing premature drug release as compared with carbopol solution alone.

The drug release pattern obtained for the gelled samples is characteristic for hydrophilic matrices. It is rapid in the begining and proceeds at a rate that declined with time. When the formulation (ISGF_3_) comes in contact with the simulated lacrimal fluid and gelation occurs, a prehydrated matrix is formed in which hydration and water penetration no longer limit drug release, leading to an apparent diffusion controlled release. In the Fig. 3, the initial fast release of TM within 15 min from undialysed gel of formulation ISGF_3_ was due to release of unentrapped drug. After 15 min the plot of cumulative percent of TM released against time upto 8 h was linear as expected for the diffusional square root of time dependence, suggesting that TM was initially released by diffusion and not through dissolution of the hydrogel. Only at later time points, 8 h and above there was indication for matrix dissolution. On the contrary the plot (Fig. 3) shows slow release of TM from dialysed gel of formulation ISGF_3_ from the beginning. It is expected that the dissolution of the gels in the cul-de-sac will proceed more slowly than that seen in the *in vitro* experiments, as the normal resident volume of the lacrimal fluid in the human eye is only 7.5–10μl. Consequently, the in vivo drug release from these systems should occur primarily by diffusion over a long duration of time, at least similar to, if not greater, than that seen in the *in vitro* experiments. The presence of the released drug in the percorneal region for long periods of time will significantly enhance the bioavailability of the drug [20].

Although the *in vitro* drug release was more sustained in case of LF_3_ as compared to ISGF_3_ (Fig 3), the ophthalmic use of the *in situ* gelling system could proved to be better in comparison to liposomes because of reduced initial rapid drainage phase of *in situ* gelling system compared to liposomes which could result in improved instilled activity remaining associated with the cornea. Nevertheless, the data indicate that the formulation ISGF_3_ might provide a considerable sustained release of TM required during the treatment of patient. Hence, *in situ* gelling system (ISGF_3_) can be adapted for the ocular delivery of TM.

### IV. Effect of variables on drug release and release kinetics

**Fig. 4.**
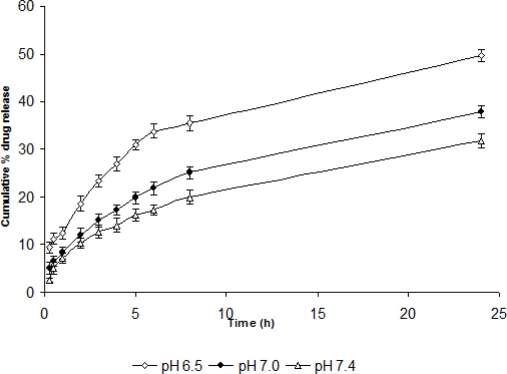
*In vitro* release of TM from dialysed gel (ISGF_3_) at different pH (n=6) (mean±sd)

**Fig. 5.**
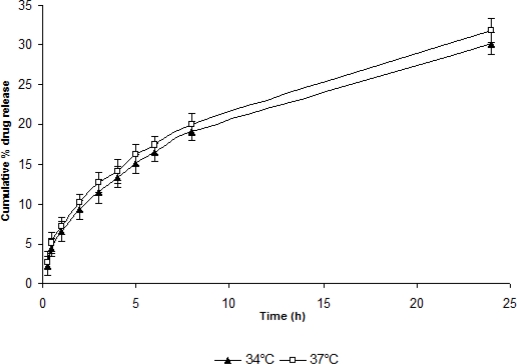
*In-vitro* release of TM from dialysed gel (ISGF_3_) at different temperatures (n=6) (mean±sd)

**Fig. 6.**
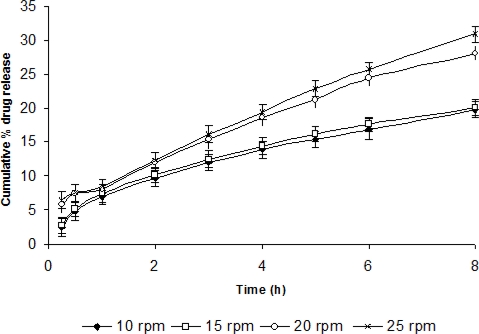
*In-vitro* release of TM from dialysed gel (ISGF_3_) at different shearing (n=6) (mean±sd)

For determining the effect of process variables like pH, temperature and shearing on drug release and release kinetics, *in vitro* drug release studies were performed using STF having different pH, temperature and shearing. As the pH was increased to 7.4, the drug release was also found to become slower i.e., sustained release was attained. Similarly, as the temperature was increased, the percent cumulative drug release was recorded to be increased. However, there was no profound effect of shearing on drug release at 10 and 15 rpm but percent drug release was found to be increased at 20 and 25 rpm probably due to erosion of gel at high shear (Fig. 4, 5, 6).

The marginal variation in the value of release index (n) at different pH (Table 3) was probably due to pH dependent solubility of the carbopol. At pH 7.4, it was more soluble, highly ionized, formed very strong cross-links with chitosan (insoluble above pH 6.2) and acted as hydrophilic matrix which showed fickian diffusion release kinetics. At lower pH, it became less soluble, showed less cross-linking with chitosan and acted as an inert matrix (due to improper gel formation) which showed both erosion as well as diffusion-controlled release.

At temperatures 37°C and 34°C, formulation showed normal fickian and approximately fickian release kinetics respectively (Table 3). Similarly at 10 and 15 rpm the formulation showed normal fickian diffusion but at 20 and 25 rpm it showed anomalous kinetics. This was probably due to erosion of the polymeric matrix at high shear.

### V. In vivo studies

**Fig. 7.**
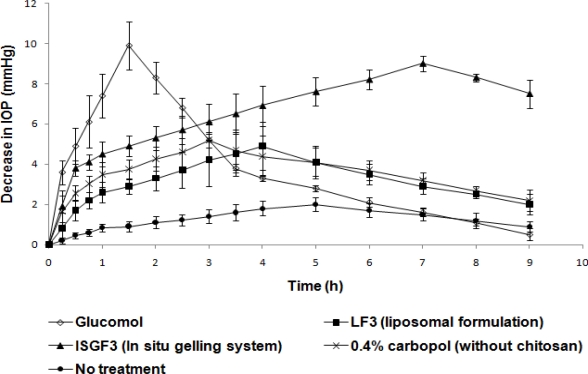
Effect of different formulations on induced IOP (n=6) (mean±sd)

*In vivo* efficacy of TM *in situ* gelling system ISGF_3_ in reducing elevated IOP (Table 4) on instilling the dosage form into eye is compared to that of LF_3_, 0.4% w/v carbopol solution (without chitosan) and Glucomol (conventional eyedrop), in Fig. 7.

Fig. 7 showed that the time corresponding to peak biological response was significantly delayed (p<0.05) by about 7 h in case of *in situ* gelling system, when compared to liposomes (4 h), 0.4% w/v carbopol solution (without chitosan) (3 h) and Glucomol (1.5 h). This suffices the very basic objective of controlled ocular delivery i.e. slow onset of action followed by an intense biological response over a prolonged period of time.

The prolonged residence of TM in the cul-de-sac, when administered in the form of ISGF_3_ formulation, increased the bioavailability of this drug, thus its therapeutic effect. This could lead to a reduction in the number of TM dosing, from 4 to only 2 times a day, contributing to increased patient compliance, in addition to the easy instillation of a liquid and reduced side effects.

The ocular bioavailability assessed in terms of the pharmacological response was calculated as AUC values (Table 5). *In situ* gelling system showed an increased bioavailability calculated in relative terms i.e. formulation ISGF_3_ showed 2.481 fold increased bioavailability over Glucomol. Formulation ISGF_3_ was found to demonstrate an AUC of 60.425 mm Hg/h, which was significantly high (p<0.05) as compared to Glucomol (24.35 mm Hg/h). The relative biological response was thus 248.1% (Table 5).

AUC values obtained following different treatments were treated statistically and the treatments were compared against Glucomol treatment. Amongst the population, variations were noted and student *t* values were determined. It was found that there was a significant difference between the AUC’s compared (p<0.05) (Fig. 7). This is the indicative of better therapeutic effectiveness of liposomal preparation over plain drug solution where as *in situ* gelling system-based treatment was found to be the best.

The enhanced and sustained peak biological response proved a prolonged contact of gel with the corneal surface as compared to liposomes, 0.4% w/v carbopol solution (without chitosan) and Glucomol. This avoided the systemic drainage of drug through nasolacrimal duct. It was also appeared that the gel was strong enough to withstand the shear forces in the cul-de-sac and demonstrated long residence times in the eye.

**Fig. 8.**
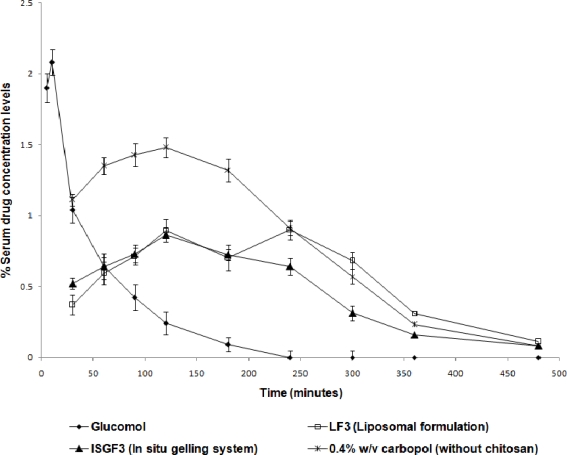
Percent TM detected in serum from different formulations (n=6) (mean±sd)

The amount of drug detected in serum after instillation of the various dosage forms into cul-de-sac was also measured. The amount of drug leaked was measured to be 2.08% after 10 min in case of Glucomol. TM being a non-selective β blocker produces serious side effects of bronchospasm and cardiovascular effects after repeated administration of eye drop. Liposomes (LF_3_) and 0.4% w/v carbopol solution (without chitosan) provided some control over the drainage as 0.893% and 1.48% of TM respectively, was measured to be drained after 2h. Whereas, only 0.862 % of drug was measured to be leaked from formulation ISGF_3_ into the circulation after 2 h, which was significantly (p<0.05) less, when compared to the conventional Glucomol eyedrop (Fig. 8).

Carbopol/chitosan eye drops showed tremendous ocular tolerance. After administration of ISGF_3_ formulation to rabbit eyes, twice daily for 7 days, no ocular damage or abnormal clinical signs to cornea, iris and conjunctiva were visible.

## Experimental

### I. Materials

Timolol maleate was obtained as a gift sample from Nicholas Piramal Limited, India. Carbopol^®^ 940 and chitosan (low viscosity grade and a degree of deacetylation ∼85%) were gifted by Panacea Biotech, India. Materials used in preparation of liposomes were soya phosphatidylcholine (PC) (Panacea Biotech, India), cholesterol (CH) (Hi Media, India) and chloroform (E Merck, India). Glucomol^®^, containing 0.25% w/v TM (IP) was purchased from Allergan Limited, India.

### II. Preparation of formulations

For the preparation of *in situ* gelling system, chitosan solution (0.5% w/v) was prepared by dissolving 50 mg of chitosan in 10 mL of acetate buffer (pH 4.6). Aqueous solutions of varying percentage of carbopol (0.2, 0.3, 0.4 and 0.5% w/v) were prepared by dispersing the required amount in double distilled water with continuous stirring (Remi instruments, India) until completely dissolved. These carbopol solutions (0.2, 0.3, 0.4 and 0.5% w/v) were mixed with equal quantity of chitosan solution (0.5% w/v) separately with continuous stirring and labeled as ISGF_1_, ISGF_2_, ISGF_3_ and ISGF_4_, respectively. These mixed polymeric solutions were sterilized by autoclaving at 121° C for 20 min. TM solution was prepared separately in distilled water aseptically and sterilized by passing through 0.2 μ membrane filter (sartorius, Germany). This solution was mixed into above polymeric solutions (ISGF_1_, ISGF_2_, ISGF_3_ and ISGF_4_) under aseptic conditions to give the final dose of 0.25% w/v. These formulations were also terminally sterilized by UV irradiation. Prepared formulations were evaluated for gelling capacity and viscosity in order to identify the compositions suitable for use as *in situ* gelling systems (Table. 1). The gelling capacity was determined by placing a drop of the system in a vial containing 2 ml of STF freshly prepared and equilibrated at 37°C and visually assessing the gel formation under microscope and noting the time for gelation and the time taken for the gel to dissolve back into solution [13]. The composition of STF used was sodium chloride 0.670 g, sodium bicarbonate 0.200 g, calcium chloride.2H_2_O 0.008 g, double distilled water q.s. 100 g. The viscosity was measured using a Brookfield Synchrolectric Viscometer (RVT model) in the small volume adaptor. The viscosity measured at 20 rpm was used for the purpose of comparative evaluation.

For comparative purpose, liposomal formulations were also prepared by conventional cast film method under aseptic conditions. Soya phosphatidyl choline and cholesterol in different molar ratios (9:1, 8:2, 7:3, 6:4 and 5:5) were dissolved by using minimum amount of chloroform (3 ml) in a round bottom flask and labeled as LF_1_, LF_2_, LF_3_, LF_4_ and LF_5_, respectively. A film of lipid was casted on the bottom of flask by evaporation of chloroform through rotation of the flask. The dried lipid film was then allowed to dry further so as to ensure complete removal of organic solvent. It was subsequently hydrated with 3 ml of 0.75% w/v aqueous solution of TM for one hour in order to form TM loaded liposomes containing TM equivalent to 0.25% w/v. Carbopol solution (0.4%) (without chitosan) containing TM equivalent to 0.25% w/v was also prepared for comparison using the method described above.

### III. In vitro characterization

The developed formulations were primarily evaluated for clarity by visual observation against a black and white background in a well-lit cabinet, drug content by UV spectrophotometry at 295 nm (Cintra 10 UV-visible spectrophotometer, Japan), pH (Orion digital pH meter, India), sol-gel transition and sterility.

### IV. Drug content determination

For determination of drug content, 1 ml of various *in situ* gelling formulations was placed in a vial containing 5 ml of STF freshly prepared and equilibrated at 37°C and gel formation was assessed visually. As soon as whole gel body was formed, the whole formulations were taken into separate dialysis tubes. The tubes were kept in a beaker containing 50 ml of STF and formulations were dialyzed for 30 min at 50 rpm and dialysis medium was replaced with fresh quantities of STF in between so as to ensure complete removal of unentrapped drug. After removal of unentrapped drug the whole gel body of various formulations was collapsed using equal volume of 1M hydrochloric acid. Collapsed gel was filtered through whatman filter paper and finally filtrate-containing TM was analyzed spectrophotometrically at 295 nm.

For determination of entrapment efficiency of liposomes the prepared liposomes (0.2 ml) were centrifuged at 3000 rpm for 3 min through a sephadexG-50 mini-column [21]. Intact liposomes free from unentrapped drug were collected and the column was washed again with 0.2 ml of STF twice. The elutes were mixed and treated with Triton X-100 (1% v/v) which caused lyses of the bilayer to release the entrapped drug. After suitable dilutions the samples were analyzed at 295 nm.

### V. Rheological studies

The developed formulation (pH 6.0) was poured into the small sample adaptor of the Brookfield Synchrolectric Viscometer and the angular velocity was increased gradually from 10 to 100 rpm. Dial reading was recorded. The formulation was then poured into an ointment jar and the pH was raised to 7.4 by the addition of 0.5M NaOH and the dial reading was again recorded and used to calculate the viscosity [13].

### VI. In vitro drug release studies

The *in vitro* release profile of TM from developed *in situ* gelling system was studied using dialysis tube method [20]. The pure formulation (2 ml) was filled into a dialysis bag which was placed in a beaker containing 50 ml of STF (Receptor medium). The beaker was placed over a magnetic stirrer (York, India) maintained at 50 rpm at 37±1°C. Aliquots of each 2 ml volume were withdrawn at specific time intervals and replaced by an equal volume of the receptor medium. The aliquots were diluted with the receptor medium and analyzed spectrophotometrically at 295 nm (Cintra 10 UV-visible spectrophotometer, Japan). *In vitro* drug release studies were also performed for 0.4% carbopol solution containing drug (without chitosan), LF_3_ (liposomal formulations without free drug) and Glucomol in a similar manner as control experiments.

As the pH of the lacrimal fluid varies from 6.5–7.4 and temperature of the eye surface varies from 34–37°C, the effect of pH, temperature and shearing (blinking) on drug release and release kinetics of ISGF_3_ by changing the pH and temperature of STF and applying shearing at different rpm to the STF was also recorded.

The kinetics of drug release from matrix devices can be described by the following equation [22].

$$\frac{{\text{M}}_{\text{t}}}{{\text{M}}_{\infty }}=\;{\text{Kt}}^{\text{n}}$$

Where $\frac{{\text{M}}_{\text{t}}}{{\text{M}}_{\infty }}$ denotes the fraction of drug released at time t, K denotes the proportionality constant and n is the release index. When n = 0.5, drug release follows normal fickian diffusion. In contrast, zero order kinetics are indicated when n=1, where as 0.5<n<1 indicates anomalous release kinetics. For analysis of *in vitro* release kinetics of TM from the test dosage forms, the value of the release index was determined from the log (M_t_/M_∞_) vs. log (t) plots. The graphs of log percent cumulative drug release against log time were plotted at various pH, temperatures and shearing and value of n was calculated from the slopes of the lines.

### VII. In vivo studies

Male rabbits weighing between 2.5–3.0 kg were used in the study. The Institutional Animals Ethical Committee of Dr. Hari Singh Gour University, Sagar approved the study. The studies were carried out with the guidelines of Council for the Purpose of Control and Supervision of Experiments on Animals (CPCSEA), Ministry of Social Justice and Empowerment, Government of India. Left eye of each rabbit was used for test while the right eye was served as control. The formulations (50 μl) were instilled into the conjunctival sac of the test eye of the rabbits of different groups. After dosing, the lids were held together for few seconds in order to avoid loss of the dosage form. Each formulation (ISGF_3_, LF_3_, 0.4% carbopol and Glucomol) was tested in at least 6 rabbits (n=6).

### VIII. Measurements of IOP

Normal IOP of both eyes of rabbits was noted using standardized shiotz tonometer (Riester, Germany). Ocular hypertension was induced by injecting betamethasone subconjunctivally upto three weeks, each time in different sectors of the eye [23] after giving local anaesthesia using 3–4 drops of 2% w/v xylocaine solution. Baseline tonometries were obtained in hypertensive eyes during the third week of betamethasone treatment, when the ocular hypertension became sufficiently stable. After a 24 hr free interval (to avoid interference from possible corneal damage), the different formulations (50 μl) were instilled into left eye of each rabbit of respective group separately using a micropipette, while the right eye served as control. IOP was measured as a function of time in the test eye before applying the test formulations and after applying it as well as in the control eye using standardized shiotz tonometer (Riester, Germany).

Ocular hypotensive activities of *in situ* gelling system and other formulations were evaluated pharmacokinetically. The magnitude of pharmacological response was assessed from the area under curve (AUC) values by trapezoidal rule [24]. Relative magnitude of biological response (Br rel) was expressed using following equation.

$${\text{Br}}_{\text{rel}}=\left[\frac{\text{AUC}\;\text{for}\;\text{test}\;\text{formulation}}{\text{AUC}\;\text{for}\;\text{aqueous}\;\text{solution}}\right]\times 100$$

### IX. Systemic drainage of various formulations

Periodic sampling of blood was also done at a definite time interval after instillation of various formulations in continuation with IOP measurement. Blood (1.5 ml) was withdrawn from the marginal ear vein of each rabbit. Each blood sample was centrifuged at 3000 rpm for 3 minutes to separate the serum. Each serum sample was spiked with 5 μl of 20 ng/μl drug solution, treated and analyzed by high performance liquid chromatographic method using Carbon 18 as a reversed phase column (Shimadzu, Japan) [25, 26].

### X. Statistical analysis

The data were statistically processed to determine the level of significance. Standard deviation (SD) was calculated and values are given as mean ± SD. Student’s t-test was used to compare mean values of different groups. Statistical significance was designated as P<0.05.

## Conclusion

The developed carbopol/chitosan based *in situ* gelling system could effectively control the release of relatively hydrophilic drugs like timolol maleate. A considerably low drainage of TM into circulation as compared to eye drops is an added advantage in the treatment of glaucoma. The formulation is also found to be suitable for sustained topical drug delivery to eyes for rational drug therapy in case of various ocular diseases. Moreover, this new formulation is a viable alternative to conventional eye drops by virtue of its ability to enhance bioavailability through its longer pre-corneal residence time and ability to sustain drug release. Also important is the ease of administration afforded and decreased frequency of administration resulting in better patient acceptance. In order to maximize the potential of this system for ocular drug delivery, further studies are needed to examine its feasibility for the prolonged delivery of other ocular drugs.

## Figures and Tables

**Tab. 1.** Percent drug content of *in situ* gelling formulations with different concentrations of carbopol and chitosan (n=6) (mean±S.D.)

**Formul. code**	**Concentr. (% w/v)**		**Clarity**	**Gelling capacity at**		**Viscosity (cp) (20 rpm)**	**% Drug content**
	Carbopol	Chitosan		**non phys. pH**	**phys. pH**		
ISGF_1_	0.2%	0.5%	Clear	−	+	342.0	42.1±3.2
ISGF_2_	0.3%	0.5%	Clear	−	++	750.0	59.5±2.1
ISGF_3_	0.4%	0.5%	Clear	−	+++	1045.0	73.2±5.2
ISGF_4_	0.5%	0.5%	Turbid	+	+++	2765.0	83.1±5.2

− No gelation; + Gels after a few minutes remains for few hours; ++ Gel after few seconds, remains for few hours; +++ Gelation immediate, remains for extended period.

**Tab. 2.** Entrapment efficiency of liposomal formulations with different molar ratio of PC and cholesterol (n=6) (mean±S.D.)

**S. No.**	**Formulation code**	**Molar ratio PC: Cholesterol**	**% Entrapment**
1.	LF_1_	9:1	27.8±2.6
2.	LF_2_	8:2	29.7±1.7
3.	LF_3_	7:3	33.2±2.4
4.	LF_4_	6:4	25.6±2.2
5.	LF_5_	5:5	24.9±1.5

**Tab. 3.** Influence of various parameters on drug release kinetics of dialysed gel (ISGF_3_) (n=6)

**Parameters**	**Release index (n)**	**Drug release kinetics**
pH		
6.5	0.55	Anomalous release kinetics
7.0	0.526	Approximately fickian
7.4	0.5	Normal fickian diffusion
Temperature		
34°C	0.52	Approximately fickian
37°C	0.5	Normal fickian
Shearing		
10 rpm	0.5	Normal fickian
15rpm	0.5	Normal fickian
20rpm	0.64	Anomalous release kinetics
25rpm	0.68	Anomalous release kinetics

**Tab. 4.** IOP values before and after injecting betamethasone (n=6) (mean±S.D.)

**IOP mm Hg**	**Left eye**	**Right eye (control)**
Normal	20.3±0.8	20.7±0.6
After injecting betamethasone upto three weeks	27.1±0.5	21.8±0.3

**Tab. 5.** AUC values of formulations expressed as decrease in IOP with respect to time (n=6) (mean±S.D.)

	**No treatment**	**Glucomol**	**LF**_**3**_	**0.4 % w/v Carbopol solution**	**ISGF**_**3**_
AUC mm Hg/h	13.8±1.9	24.35±3.5	29.2±2.5	41.8±1.8	60.425±3.2
BR_rel_	−	−	1.199 folds	1.71 folds	2.481 folds

## References

[1] Sieg JW, Robinson JR (1976). Mechanistic studies of transcorneal permeation of Pilocarpine. J Pharm Sci.

[2] Lee VHL, Mitra AK (1993). Precorneal, corneal and postcorneal factors. Ophthalmic Drug Delivery Systems, Drug and the Pharmaceutical Sciences.

[3] Chein YW, Cabana BE, Mares SE, Chein YW (1982). Ocular controlled-release drug administration. Novel Drug Delivery Systems: Fundamentals, Developmental Concepts, Biomedical Assessments, Drugs and the Pharmaceutical Sciences.

[4] Schoenwald RD, Smolen VF (1971). Drug absorption analysis from pharmacological Data II: Transcorneal biophasic availability of tropicamide. J Pharm Sci.

[5] Lee VHL, Robinson JR (1986). Topical ocular drug delivery: Recent developments and future challenges. J Ocular Pharmacol.

[6] Lee VHL (1990). New directions in the optimization of ocular drug delivery. J Ocular Pharmacol.

[7] Munroe WP, Rindone JP, Kershner RM (1985). Systemic side effects associated with the ophthalmic administration of timolol. Drug Intell Clin Pharm.

[8] Urtti A, Salminen L (1993). Minimizing systemic absorption of topically administered ophthalmic drugs. Surv Ophthalmol.

[9] Nelson WL, Fraunfelder FT, Sills JM (1986). Adverse respiratory and cardiovascular events attributed to timolol ophthalmic solution. Am J Ophthalmol.

[10] Davies NM, Farr SJ, Hadgraft J, Kellaway IW (1991). Evaluation of mucoadhesive polymers in ocular drug delivery. I. Viscous solutions. Pharm Res.

[11] Chenite A, Chaput C, Wang D, Combes C, Buschmann MD, Hoemann CD, Leroux JC, Atkinson BL, Binette F, Selmani A (2000). Novel injectable neutral solutions of chitosan form biodegradable gels *in situ*. Biomaterials.

[12] Schoenwald RD, Ward RL, DeSantis LM, Roehrs RE (1978). Influence of high viscosity vehicles on miotic effect of pilocarpine. J Pharm Sci.

[13] Srividya B, Cardoza RM, Amin PD (2001). Sustained ophthalmic delivery of ofloxacin from a pH triggered *in situ* gelling system. J Control Rel.

[14] Kumar S, Himmelstein KJ (1995). Modification of in situ gelling behavior of carbopol solutions by hydroxypropyl methylcellulose. J Pharm Sci.

[15] Miller SC, Donovan MD (1982). Effect of Poloxamer 407 gel on the mitotic activity of pilocarpine nitrate in rabbits. Int J Pharm.

[16] Vadnere M, Amidon G, Lindenbaum S, Haslam JL (1984). Thermodynamic studies on the gel-sol transition of some pluronic polyols. Int J Pharm.

[17] Gurny R, Boye T, Ibrahim H (1985). Ocular therapy with nanoparticulate systems for controlled drug delivery. J Control Rel.

[18] Rozier A, Manuel C, Grove J, Plazonnet B (1989). Gelrite^®^: A novel, ion-activated, *in-situ* gelling polymer for ophthalmic vehicles. Effect on bioavailability of timolol. Int J Pharm.

[19] Joshi A, Ding S, Himmelstein KJ (1993). Reversible gelation composition and methods of use.

[20] Cohen S, Lobel F, Trevgoda A, Peled Y (1997). A novel in situ-forming ophthalmic drug delivery systems from alginates undergoing gelation in the eye. J Control Rel.

[21] New RRC, New RRC (1990). Introduction and preparation of liposomes. Liposomes: A practical approach.

[22] Sanzgiri YD, Maschi S, Crescenzi V, Callegaro L, Topp EM, Stella VJ (1993). Gellan based systems for ophthalmic sustained delivery of methylprednisolone. J Control Rel.

[23] Bonomi L, Perfetti S, Noya E, Bellucci R, Tomazzoli L (1978). Experimental corticosteroid ocular hypertension in the rabbit. Graefes Arch Klin Exp Ophthalmol.

[24] Rowland M, Tozer TN (1995). Assessment of AUC. Clinical Pharmacokinetics, Concepts and Applications.

[25] Kubota K, Yamada T, Ogura A, Ishizaki T (1990). A novel differentiation method of vehicle models for topically applied drugs: Application to a therapeutic timolol patch. J Pharm Sci.

[26] Kubota K, Koyama E, Yasuda K (1991). Skin irritation induced by topically applied timolol. Br J Clin Pharmacol.

